# Effects of Melatonin on the Proliferation and Apoptosis of Sheep Granulosa Cells under Thermal Stress

**DOI:** 10.3390/ijms151121090

**Published:** 2014-11-14

**Authors:** Yao Fu, Chang-Jiu He, Peng-Yun Ji, Zhi-Yong Zhuo, Xiu-Zhi Tian, Feng Wang, Dun-Xian Tan, Guo-Shi Liu

**Affiliations:** 1 Key Laboratory of Animal Genetics and Breeding of the Ministry of Agriculture, National Engineering Laboratory for Animal Breeding, Beijing Key Laboratory for Animal Genetic Improvement, National Key Laboratory of Animal Nutrition, College of Animal Science and Technology, China Agricultural University, Beijing 100193, China; E-Mails: yokofu2005dwx@126.com (Y.F.); chungjoy@cau.edu.cn (C.-J.H.); jipengyun1989@126.com (P.-Y.J.); zzy_8702@163.com (Z.-Y.Z.); tian7550@163.com (X.-Z.T.); vicent007@126.com (F.W.); 2 College of Animal Science, Jilin University, Changchun 130062, China; 3 College of Animal Science, Xinjiang Agricultural University, Wulumuqi 830052, China; 4 Department of Cellular & Structural Biology, The UT Health Science Center, San Antonio, TX 78229, USA; E-Mail: tan@uthscsa.edu

**Keywords:** melatonin, sheep, granulosa cell, proliferation, apoptosis, thermal stress, antioxidant

## Abstract

The cross-talk between oocyte and somatic cells plays a crucial role in the regulation of follicular development and oocyte maturation. As a result, granulosa cell apoptosis causes follicular atresia. In this study, sheep granulosa cells were cultured under thermal stress to induce apoptosis, and melatonin (MT) was examined to evaluate its potential effects on heat-induced granulosa cell injury. The results demonstrated that the Colony Forming Efficiency (CFE) of granulosa cells was significantly decreased (heat 19.70% ± 1.29% *vs.* control 26.96% ± 1.81%, *p < 0.05*) and the apoptosis rate was significantly increased (heat 56.16% ± 13.95%*vs.* control 22.80% ± 12.16%, *p < 0.05*) in granulosa cells with thermal stress compared with the control group. Melatonin (10^−7^ M) remarkably reduced the negative effects caused by thermal stress in the granulosa cells. This reduction was indicated by the improved CFE and decreased apoptotic rate of these cells. The beneficial effects of melatonin on thermal stressed granulosa cells were not inhibited by its membrane receptor antagonist luzindole. A mechanistic exploration indicated that melatonin (10^−7^ M) down-regulated *p53* and up-regulated *Bcl-2* and *LHR* gene expression of granulosa cells under thermal stress. This study provides evidence for the molecular mechanisms of the protective effects of melatonin on granulosa cells during thermal stress.

## 1. Introduction

Thermal stress disrupts spermatogenesis, follicle development, oocyte maturation, early embryonic development, fetal and placental growth and lactation. It has negative impacts on human health and also causes serious problems in the livestock industry [1]. The effects of high temperature on gametes and early embryos may involve an increased production of reactive oxygen species (ROS). Under physiological conditions, ROS formation and elimination is a dynamic balance. Thermal stress disturbs this balance and promotes ROS production in cells, which, in turn, causes cellular oxidative stress [2]. These effects include DNA, protein and lipid damage, which ultimately leads to cell apoptosis or necrosis [3,4]. Melatonin (MT), a tryptophan derivative first identified in the pineal gland of vertebrates, has an important role in the control of seasonal reproduction in photoperiodic animals, the promotion of sleep in some species and the regulation of body temperature [5,6]. In addition, melatonin also functions as an anti-tumor and anti-aging agent and provides protective effects for the gastrointestinal and cardiovascular systems [7,8,9]. In the peripheral reproductive organs, melatonin maintains normal physiology and functional integrity [10,11]. It is widely believed that the membrane receptors MT1/MT2, the cytosolic binding site MT3 and the nuclear receptor ROR partially mediate the physiological functions of melatonin, MT1 and MT2, which are primarily located on cells of the pituitary pars tuberalis (PT) and suprachiasmatic nucleus (SCN) and distributed in peripheral tissues [12,13,14]. Nevertheless, recent research has provided evidence that ROR is not a receptor for melatonin [15].

Melatonin is a potent free radical scavenger and antioxidant [16]. Because it is amphiphilic, melatonin can reach any cellular compartment, including the membrane, cytosol and mitochondria, with ease. Importantly, it inhibits peroxidation, which is a common feature of other antioxidants. Regarding the free radical scavenging capacity, melatonin is 5-fold more potent than glutathione (GSH) and 8-fold more potent than mannitol [17]. In addition, the anti-stress effects of melatonin on hypoxia, burning injury, noise, and light disturbance have been extensively studied [18,19,20,21,22,23,24,25,26,27]. The results demonstrate that melatonin effectively protects organisms against theses environmental insults. The anti-stress activity of melatonin, at least in part, contributes to its anti-aging and health beneficial effects under adverse circumstances [28,29]. The role of melatonin in cell proliferation and apoptosis are cell type dependent [30,31]. In tumor cells, such as human hepatoma, breast cancer, osteosarcoma and neural tumor cells, melatonin inhibits cell proliferation and promotes apoptosis [32,33]. In contrast, it stimulates the proliferation, differentiation and maturation of a variety of normal cells, including human bone cells and rat embryonic neural stem cells [34]. In mesencephalic neural stem cells (NSCs), MT stimulates the proliferation and differentiation of dopaminergic neurons and inhibits their differentiation to astrocyte cells [35].

Little is known regarding the effects of MT on cellular proliferation and apoptosis in reproductive supporting cells. In the current study, sheep granulosa cells were used to address this question.

Granulosa cells are the somatic cells that surround oocytes. In mammals, oocytes undergo a prolonged and carefully regulated developmental process as a result of instructive paracrine and junctional interactions with granulosa cells [36]. It has been demonstrated that follicular selection and atresia depend on granulosa cell apoptosis [37,38]. The aim of this study was to explore the effects and mechanisms of melatonin on granulosa cell proliferation and apoptosis under thermal stress. The results will provide basic knowledge regarding the role of melatonin in follicular development and atresia-related functions.

## 2. Results

### 2.1. Effects of Melatonin on the Cloning Efficiency of Granulosa Cells

**Figure 1:**
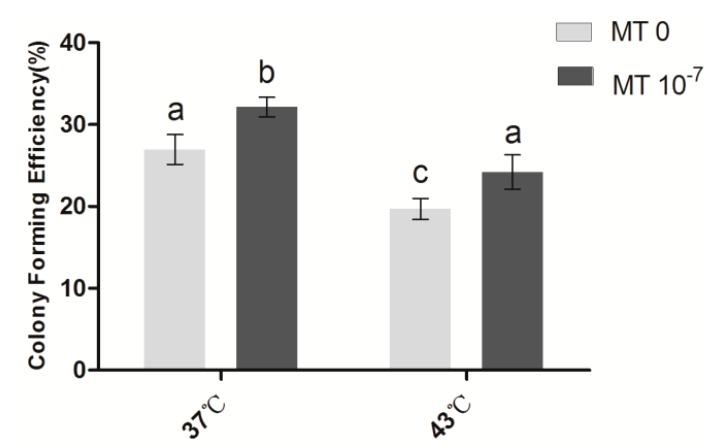
Effects of melatonin on the CFE of granulosa cells. MT: melatonin. Each bar represents the mean ± SEM for experiments performed in triplicate. Different letters indicate significant differences, *p* < 0.05.

As shown in Figure 1, the colony forming efficiency (CFE) of sheep granulosa cells in the group with thermal stress (43 °C) (19.7% ± 1.29%) was significantly lower than the control group (37 °C) (27.0% ± 1.81%) (*p* < 0.05). Following melatonin (10^−7^ M) treatment, the CFEs of both the thermal stressed and control groups were significantly increased compared with their melatonin-free counterparts (*p* < 0.05).

### 2.2. Effects of Melatonin on Granulosa Cell Apoptosis

**Figure 2:**
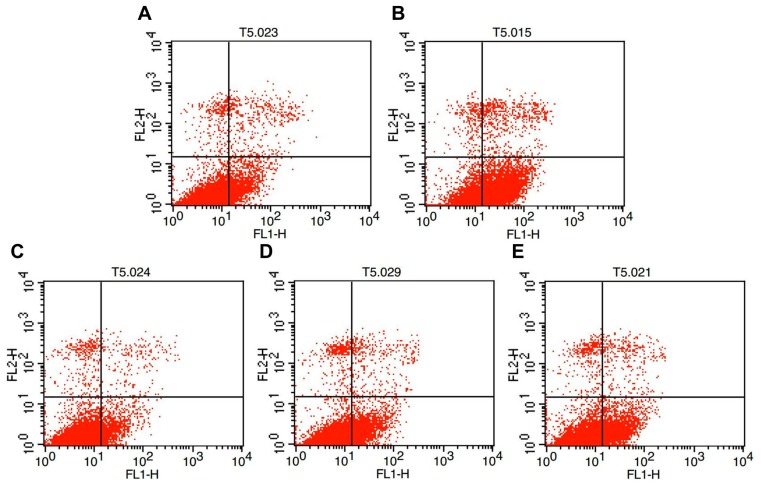
Low cytometry analysis of cell apoptosis. (**A**) Control cells (37 °C); (**B**) Control cells (37 °C) with MT (10^−7^ M); (**C**) Thermal stressed cells (43 °C) without MT; (**D**) Thermal stressed cells (43 °C) with MT (10^−7^ M); (**E**) Thermal stressed cells (43 °C) with MT(10^−7^ M) and Luzindole (10^−6^ M). The Upper Left Quadrant: necrotic cells; The Upper Right Quadrant: late apoptotic cells; The Lower Left Quadrant: normal cells; The Lower Right Quadrant: early apoptotic cells.

**Figure 3:**
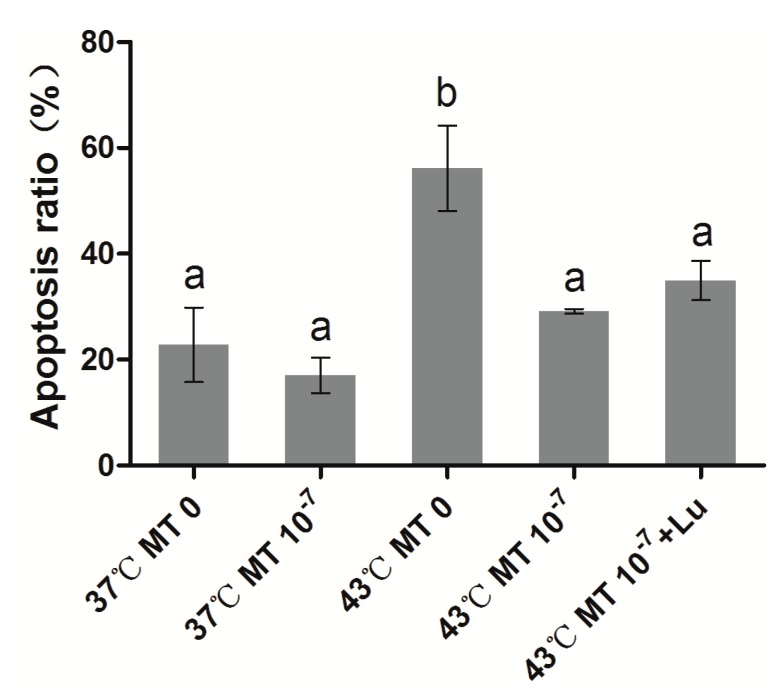
Effects of melatonin on granulosa cell apoptosis. MT: melatonin; Lu: luzindole. Each bar represents the mean ± SEM for experiments performed in triplicate. Different letters indicate significant differences, *p* < 0.05.

As shown in Figure 2 and Figure 3, the percentage of apoptotic granulosa cells in the groups subjected to thermal stress (43 °C) (56.2% ± 13.94%) was significantly higher than the control group (37 °C) (22.8% ± 12.16%) (*p* < 0.05). The percentage of apoptotic cells in the thermal stressed group with 10^−7^ M melatonin was significantly lower than the group without melatonin, and it was not significantly different (*p* > 0.05) from the control group. It appears that the melatonin receptor antagonist luzindole does not influence the antiapoptotic effects of melatonin on granulosa cells under thermal stress.

### 2.3. Effects of Melatonin on the Expression of Apoptosis Genes in Sheep Granulosa Cells

**Figure 4:**
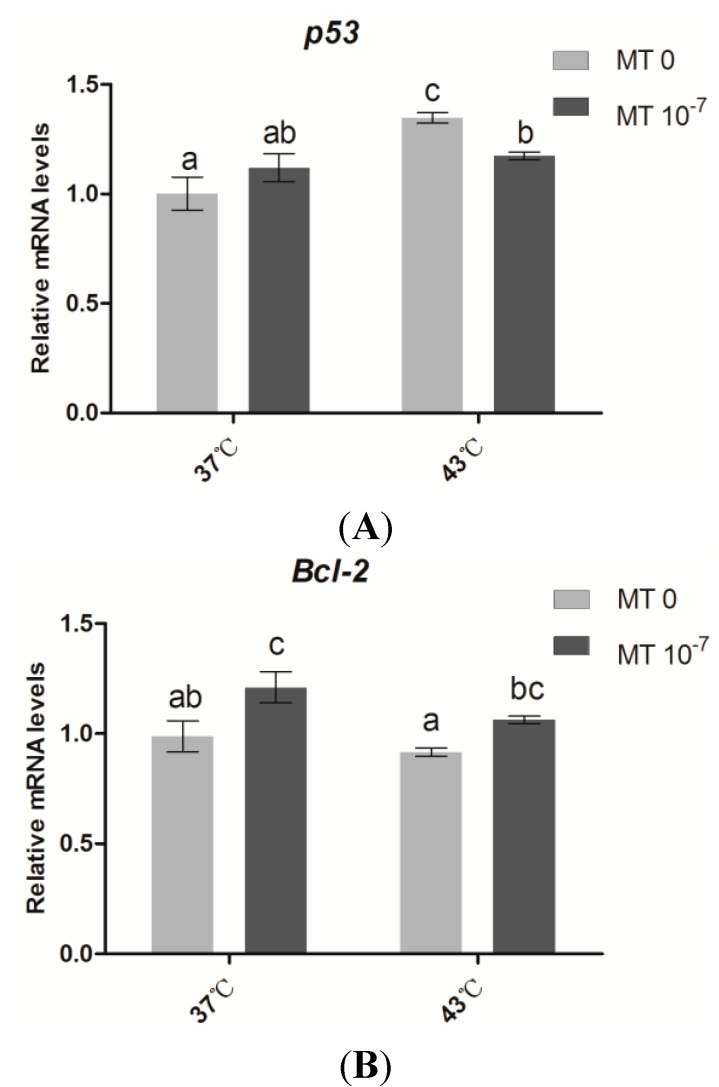
Effects of melatonin on the relative expression levels of *p53* and *Bcl-2* in sheep granulosa cells. (**A**) The relative expression of *p53* at different treatments; (**B**) The relative expression of *Bcl-2* at different treatments. MT: melatonin. Each bar represents the mean ± SEM for experiments performed in triplicate. Different letters in the same column represent significant differences, *p* < 0.05.

As shown in Figure 4, the expression level of *p53* in the sheep granulosa cells under thermal stress (43 °C) was significantly higher than the control group (37 °C); however, *Bcl-2* gene expression was not significantly different between the thermal stressed and control groups. The expression level of *p53* was significantly lower in the thermal stressed group treated with melatonin (10^−7^ M). The phenomenon in *Bcl-2* expression was not similar to *p53*. The expression level of *Bcl-2* treated with melatonin (10^−7^ M) was significantly increased not only in the group at 37 °C but also in the 43 °C treated group.

### 2.4. Effects of Melatonin on the Gene Expression of the Gonadotropin Receptor LHR in Sheep Granulosa Cells

**Figure 5:**
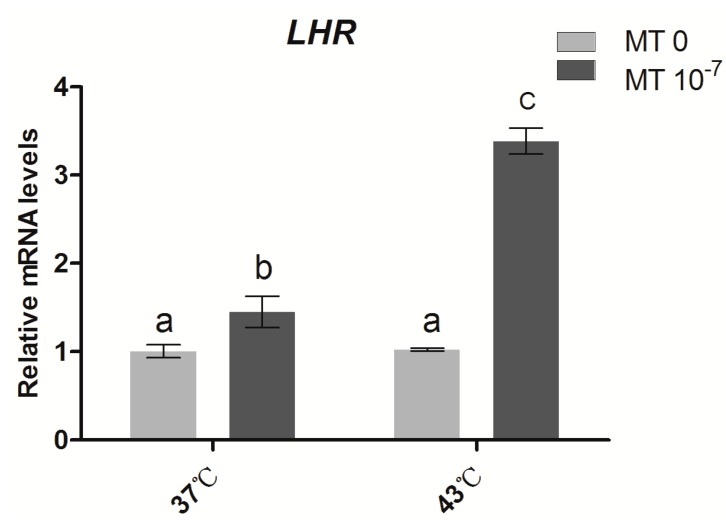
Effects of melatonin on the relative expression of *LHR* in sheep granulosa cells MT: melatonin. Each bar represents the mean ± SEM for experiments performed in triplicate. Different letters in the same column represent significant differences, *p* < 0.05.

As shown in Figure 5, under thermal stress (43 °C), the mRNA expression level of *LHR* in granulosa cells was not significantly different from the control groups (37 °C). When cells were incubated at 37 °C and supplemented with melatonin (10^−7^ M), the *LHR* expression level was higher than the controls. Moreover, a significant increase in *LHR* gene expression was observed in the thermal stressed groups treated with melatonin (10^−7^ M).

## 3. Discussion

Antioxidants play a protective role against oxidative damage caused by thermal stress in the cells and tissues of organisms. The positive effects of antioxidants have been reported regarding several reproductive aspects [39,40,41,42,43,44,45,46,47,48] and the recovery of injuries induced by thermal stresses [49,50]. Melatonin treatment for high-yielding dairy cows during a dry period under thermal stress improved their reproductive performance and reduced the rates of breeding syndrome and pregnancy loss [51,52].

In physiological conditions, cells can maintain their dynamic balance of ROS production and elimination. In contrast, thermal stress can disrupt this balance and lead to oxidative damage in cells. In this study, we observed that thermal stress significantly reduced the CFE and elevated the apoptosis rate in sheep granulose cells. These results are consistent with other findings in mouse [52]. It has been observed that melatonin at the appropriate concentration (10^−4^ M) promoted bovine blastocyst development [53]. Several recent studies have shown that melatonin promotes oocyte maturation and embryo development in the mouse, bovine and porcine. When a culture medium of porcine and mouse embryos was supplemented with melatonin (10^−7^ M), the cleavage rate, blastocyst rate and cell number of blastocytes were significantly increased [54,55,56,57]. More importantly, melatonin(10^−7^ M) reduces ROS production and cellular apoptosis during *in vitro* embryo development and improves the quality of blastocysts, up-regulates the relative expression of the antioxidant enzyme superoxide dismutase (SOD) and the anti-apoptotic factor *Bcl-2* and down-regulates the pro-apoptotic gene *p53* [55]. Based on these previous reports, 10^−7^ M melatonin was selected as the optimal concentration in the current study. It was observed that melatonin at this concentration (10^−7^ M) significantly increased the CFE and decreased the apoptotic rate of sheep granulosa cells caused by thermal stress (43 °C) (Figure 1). These results suggest that melatonin plays an important role in the protection of sheep granulosa cells from the harmful effects caused by thermal stress and this protection is most likely related to its antioxidant capacity.

It is well-known that in apoptosis, cytochrome C (cytC) released from mitochondria binds to Apaf-1 (a cytoplasmic protein that contains a caspase binding domain). This combination increases the binding affinity of Apaf-1 to dATP/ATP. dATP/ATP then binds to the cytC/Apaf-1 complex and forms a programmed death body (apoptosome); apoptosome further activates downstream factors through enzyme digestion to guide programmed cell death [58]. The release of cytC is suppressed by *Bcl-2*, an important member of the anti-apoptotic family. *Bcl-2* plays a critical role in the regulation of antral follicle atresia. *Bcl-2* knockout animals have a reduced number of healthy follicles, and local over-expression of the *Bcl-2* gene in the granulosa cells of developing follicles decreases apoptosis [59,60]. Melatonin has been reported to inhibit the release of cytC from mitochondria, and thereby reduces apoptosis in neural hippocampal cells [61,62]. In the current study, we observed that the *Bcl-2* expression level of granulosa cells under thermal stress was significantly up-regulated by melatonin treatment. This finding was consistent with the results previously discussed [55,56]. Thus, we speculate that melatonin may directly regulate *Bcl-2* and subsequently inhibit cytC release from mitochondria. We also recognized that *p53* is another important factor in the regulation of *Bcl-2*. In general, *p53* is regarded as a key player in tumor suppression because it promotes growth arrest, apoptosis and cellular senescence. Most importantly, the phosphorylation sites on *p53* are Ser-15, which promotes accumulation and activation of *p53* and DNA repair, and Ser-46, which regulates apoptosis following DNA damage. The former can be up-regulated by melatonin in a stress-induced system [63]. *p53* also has the ability to regulate the transcription of various apoptotic genes, including the Bcl-2 family. The inhibition of *p53* expression can up-regulate *Bcl-2* proteins in a rat model of cholestasis [64,65,66,67,68]. In this study, *p53* expression, which was elevated by thermal stress in sheep granulose cells, was significantly reduced by melatonin treatment (Figure 4). The results also suggested that *p53* participated in the anti-apoptotic function of melatonin via the *Bcl-2* pathway [56].

Luteinizing hormone (LH) is an important hormone in the differentiation process of granulosa cells, and it regulates the development process of preantral follicles to ovulation follicles. It is an obligatory step in the differentiation and maturation of granulosa cells and is also essential for the initiation of luteinization [69,70,71,72]. It was demonstrated that during follicular atresia and granulosa cell apoptosis, LH receptors in the ovary significantly decreased. When follicles or granulosa cells were treated with FSH or LH, it inhibited follicular atresia and cell apoptosis [73,74]. A recent study has shown that melatonin treatment significantly increased the mRNA expression of the LH receptor but not of FSH; furthermore, melatonin was thought to be involved in maintaining the appropriate level of *LHR* expression for ovarian function [11,75,76,77]. Similar results were observed in the current study. As shown in Figure 5, the expression of *LHR* in granulosa cells under thermal stress was not significantly different from the control. This finding suggests that *LHR* gene expression is not significantly affected by increased temperature. However, melatonin treatments remarkably up-regulated the expression level of *LHR* in both control and thermal stressed groups. It appears that melatonin can induce the expression of the *LHR* gene, which thereby improves the quality of granulosa cells and enhances their ability to protect against thermal-stress.

In conclusion, melatonin at 10^−7^ M was demonstrated to effectively protect sheep granulosa cells from the harmful effects caused by thermal stress. This effect is indicated by an increase in the formation of CFE and a decrease in the apoptotic rate. The anti-apoptotic effects of melatonin in thermal stressed granulose cells are primarily attributed to its activities that down-regulated *p53* and up-regulated *Bcl-2* and *LHR* gene expression. These effects of melatonin may involve its antioxidant capacity, since many naturally occurring antioxidants exhibit similar functions.

## 4. Materials and Methods

### 4.1. Materials

DMEM, FBS and TCM199 were products of GIBCO Company (Carlsbad, CA, USA). Trypsin and PBS were purchased from Beijing Maichen Technology Company (Beijing, China). An Annexin V-FITC Apoptosis Assay Kit was obtained from Beyotime Institute of Biotechnology (Beijing, China). Melatonin and all other chemicals were of the highest analytical and tissue culture grades and were purchased primarily from Sigma Aldrich Chemical Company (St. Louis, MO, USA). The sheep ovaries were collected from the local abattoir.

### 4.2. Granular Cell Separation and Culture

Adult ovine ovaries were collected, stored in physiological saline and transported to the laboratory within 3–4 h. The ovaries were cleaned repeatedly with physiological saline that contained antibiotics. The follicles were cut to a 2–6 mm size in diameter using a surgical knife blade. Granule cells were aspirated from the follicle fluid and washed with Dulbecco’s phosphate-buffered saline three times. The suspended cells were cultured with DMEM/F12 that contained 10% FBS in the cell culture plate. They were incubated at 37 °C with 5% CO_2_ in humidified air.

### 4.3. Measurement of Colony Forming Efficiency

The granular cells were divided into normal temperature (37 °C) and thermal stressed groups (43 °C). MT was added to the medium with a final concentration of 10^−7^ M. After the cells adhered, in the thermal stressed group, the culture temperature was increased from 37 to 43 °C with 5% CO_2_ in humidified air for 2 h; the temperature was then decreased to 37 °C. This procedure was repeated every 24 h. The colony-forming efficiency (CFE) was evaluated on the 12th day of culture. The colony efficiency of the isolated cells was evaluated by inoculating single-cell suspensions at a density of 1000 cells/well in a 10 mm cell culture plate. The cells were incubated in DMED/F12 medium supplemented with 10% fetal bovine serum and 1 × 10^−7^ M MT 10 mL per well. The medium was replaced every two days. Colony formation was monitored by microscopy and analyzed on day 12 after removal of the medium. The cells were fixed in methanol for 5 min and stained with 50% Giemsa staining at room temperature for 15 min. The colony-forming efficiency was calculated as the number of clones/total number of cells seeded per well.

### 4.4. Flow Cytometric Analysis of Apoptotic Cells

The granular cells were divided into 4 groups: control group (37 °C); thermal stress group (43 °C); thermal stress plus MT 10^−7^ group; thermal stress plus MT 10^−7^ and luzindole 10^−6^ group. After exposed to 43 °C for 2 h, the granulosa cells were cultured at 37 °C for 12 h; flow cytometry was subsequently performed to analyze the apoptotic cells. The apoptotic cells were differentiated from viable or necrotic cells by the combined application of annexinV-FLUOS and propidium iodide (PI). The three parallel samples were washed twice. The cells were harvested via the method of 0.25% trypsin + 0.02% EDTA and centrifuged at 1500 r/min for 5 min. The pellet was re-suspended and washed twice with cold PBS. The cell suspension was added to 195 µL binding buffer and 5 µL Annexin V-FITC and incubated at room temperature for 10 min in darkness. The cells were centrifuged at 1500 r/min for 5 min, and the supernatant was discarded. Finally, 200 µL binding buffer that contained 10 µL PI was added to each tube. The samples were immediately analyzed using FACS (Becton, Dickinson and Company, Franklin Lake, NJ, USA).

### 4.5. RNA Isolation and Quantitative RT-PCR

Ovine granulose cells were divided into normal temperature (37 °C) and thermal stressed groups (43 °C). The thermal stressed group was incubated with melatonin (10^−7^ M) in culture medium. In the thermal stressed groups, the cells were exposed to 43 °C for 2 h; these cells were subsequently cultured at a normal temperature (37 °C) for an additional 12 h. Finally, the cells were harvested. The harvested cells were washed twice with D-PBS solution and centrifuged at 1500 rpm for 5 min. The pellet was stored at 80 °C until the RNA was extracted. The total RNA was extracted using TRIzol reagent (Invitrogen Inc., Carlsbad, CA, USA), and it was quantified by measuring the absorbance at 260 nm. The extracted RNA was stored at 80 °C until use. The levels of relevant mRNAs, including the apoptosis-related genes p53 and Bcl-2 and the gonadotropic hormone receptor LFR, were detected by quantitative RT-PCR using a One Step SYBR PrimeScript RT-PCR Kit (TaKaRa Bio., Inc., Tokyo, Japan) in a Light Cycler instrument (Roche Applied Science, Mannheim, Germany). The levels of accumulated fluorescence were analyzed using the second-derivative method after the melting-curve analysis was complete. The relative expression levels of the target genes were calculated with the 2^−ΔΔ*C*t^ method. The results were normalized to the GAPDH expression level in each sample. The primer pairs for the analyzed mRNAs are listed in Table 1.

**Table 1:** Primers used in this study.

Genes	Accession Number	Primers	Sequence (5'–3')	Product Size (bp)
*GAPDH*	HM043737	Forward	GTGTCTGTTGTGGATCTGACCTG	162
		Reverse	AGAAGAGTGAGTGTCGCTGTTGAAGT	
*p53*	FJ855223	Forward	GCACGACCATCCACTACAACTTC	148
		Reverse	GGACAGGCACAAACACGCAC	
*Bcl-2*	DQ152929	Forward	ACTTCGCCGAGATGTCCAG	138
		Reverse	CGACACCTCCGAACTCAAAG	
*LHR*	L36329	Forward	TCTGCTCACCCAAGACACTCC	247
		Reverse	GAGGCAATGAGTAGCAGGTAGAG	

### 4.6. Statistical Analysis

All data are expressed as the mean ± SEM. The data were subjected to multiple comparison analyses using GLM(General Linear Model) analysis for the intergroup comparison with SPSS 19.0 statistical software (SPSS Inc., Chicago, IL, USA). The correlations were analyzed using the Correlations procedure. *p* < 0.05 was considered statistically significant.
